# Heterogeneity in children's reading comprehension difficulties: A latent class approach

**DOI:** 10.1002/jcv2.12177

**Published:** 2023-06-05

**Authors:** Emma James, Paul A. Thompson, Lucy Bowes, Kate Nation

**Affiliations:** ^1^ Department of Experimental Psychology University of Oxford Oxford UK; ^2^ Department of Psychology University of York York UK; ^3^ Centre for Educational Development, Appraisal and Research University of Warwick Coventry UK

**Keywords:** ALSPAC, cognitive difficulties, mixture models, multiple deficits, poor comprehenders, reading development

## Abstract

**Background:**

Poor comprehenders are traditionally identified as having below‐average reading comprehension, average‐range word reading, and a discrepancy between the two. While oral language tends to be low in poor comprehenders, reading is a complex trait and heterogeneity may go undetected by group‐level comparisons.

**Methods:**

We took a preregistered data‐driven approach to identify poor comprehenders and examine whether multiple distinct cognitive profiles underlie their difficulties. Latent mixture modelling identified reading profiles in 6846 children from the Avon Longitudinal Study of Parents and Children, based on reading and listening comprehension assessments at 8–9 years. A second mixture model examined variation in the cognitive profiles of weak comprehenders, using measures of reading, language, working memory, nonverbal ability, and inattention.

**Results:**

A poor comprehender profile was not identified by the preregistered model. However, by additionally controlling for overall ability, a 6‐class model emerged that incorporated a profile with relatively weak comprehension (*N* = 947, 13.83%). Most of these children had weak reading comprehension in the context of good passage reading, accompanied by weaknesses in vocabulary and nonverbal ability. A small subgroup showed more severe comprehension difficulties in the context of additional cognitive impairments.

**Conclusions:**

Isolated impairments in specific components of reading are rare, yet a data‐driven approach can be used to identify children with relatively weak comprehension. Vocabulary and nonverbal ability were most consistently weak within this group, with broader cognitive difficulties also apparent for a subset of children. These findings suggest that poor comprehension is best characterised along a continuum, and considered in light of multiple risks that influence severity.

## Graphical abstract

We used a data‐driven approach to identify different profiles of reading ability in 6846 children from the Avon Longitudinal Study of Parents and Children. The data did not support distinct profiles of decoding and comprehension difficulties, but a continuous distribution of reading skill. An additional exploratory analysis identified children with relatively weak comprehension compared to decoding ability: the majority had weaknesses in oral language and nonverbal ability (Class 2), but broader cognitive difficulties were apparent in those with more severe comprehension problems (Class 1).

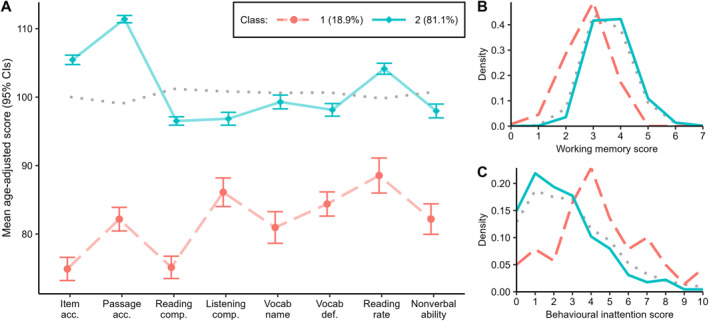

**Key points**

- Poor comprehenders show poor reading comprehension in the context of adequate decoding. While oral language tends to be low in poor comprehenders, reading is a complex trait and heterogeneity may go undetected by group‐level comparisons.
- Using a large sample and a preregistered data‐driven approach, reading comprehension difficulties were best conceptualised along a dimension of overall reading skill, rather than as a distinct subgroup.
- The majority of weak comprehenders showed weaknesses in oral language and nonverbal ability. A minority had broader cognitive weaknesses and more severe comprehension problems.
- In line with a multiple risk framework, researchers and practitioners should look beyond language to identify the broader cognitive strengths and weaknesses of poor comprehenders.

## INTRODUCTION

Poor comprehenders have difficulties with reading comprehension alongside relative strengths in reading accuracy. Their weaknesses extend beyond the written domain to listening comprehension, in line with the Simple View of Reading that sees variation in both reading accuracy (often summarized as ‘decoding’) and listening comprehension as contributing to reading comprehension (Hoover & Gough, 1990). Consistent evidence points to underlying impairments in aspects of oral language (Landi & Ryherd, 2017). However, reading comprehension is the product of many cognitive operations (Castles et al., 2018) meaning there may be several routes to comprehension failure. Large‐scale data‐driven approaches are needed to identify and understand heterogeneity.

### Oral language as a core deficit

Several studies converge on the finding that poor comprehenders have poor vocabulary relative to control children (e.g., Cain & Oakhill, 2006; Nation et al., 2004) and differences in grammatical processing and listening comprehension (Elwér et al., 2015) are indicative of oral language weaknesses more broadly. Given reciprocal influences across development (Verhoeven et al., 2011), low language may be a cause or consequence of poor reading comprehension. Retrospective longitudinal studies show that language differences are apparent *prior* to the onset of reading (Catts et al., 2006; Justice et al., 2013; Nation et al., 2010) and some causal role is further supported by the success of oral language interventions in improving reading comprehension in poor comprehenders (Clarke et al., 2010). In light of these findings, it is tempting to conclude that poor comprehenders have core deficits in oral language that lead to their difficulties with reading comprehension.

### Beyond oral language

Despite group‐level differences, not all poor comprehenders show vocabulary impairments (Colenbrander et al., 2016), and some studies find only weak transfer effects from oral language intervention to improvements in reading comprehension (Melby‐Lervåg & Lervåg, 2014). These findings align with the more general conclusion that single deficit models of developmental disorders rarely hold up to the variability observed at an individual level (Astle & Fletcher‐Watson, 2020; Pennington, 2006). Relatedly, the apparent specificity of language deficits might result from the tightly controlled group‐match design used in experimental research. Typically, small groups of children matched closely on age, reading accuracy, and often nonverbal ability (restricted to be within the normal range) are compared in attempts to identify cognitive differences that characterise poor comprehenders. By minimising sources of variability within and between the groups under investigation, however, we likely miss the breadth of weaknesses that might accompany comprehension difficulties on an individual basis. Further, such recruitment constraints typically leave small numbers of participants for the comparisons of interest (often no more than 10–20 per group), resulting in low statistical power for detecting smaller effects. Thus while language may be the most substantial difficulty experienced by the majority of poor comprehenders, milder or less consistent cognitive weaknesses may go undetected.

The proposal that poor comprehenders experience broader cognitive deficits is not novel. Reading comprehension places significant demands on other cognitive abilities, including attention (Cain & Bignell, 2014), working memory (Carretti et al., 2009), and reasoning skills, all of which may act as “pressure points” in the reading system (Logan & LARRC, 2017; Perfetti et al., 2014). While it is difficult to separate some of these effects from low language (Pimperton & Nation, 2010), documenting and understanding broader weaknesses is necessary for remediation as well as for theory. For example, mutualistic relationships between cognitive domains across development may leave long‐lasting weaknesses that are not addressed by targeting the original “cause” of the disorder in intervention (Kievit, 2020). Conversely, co‐occurring strengths in other domains may act as protective factors in minimising the severity of comprehension problems. Thus, there is a clear need for large‐scale studies that capture relative strengths and weaknesses across several dimensions, and how they might differ within a heterogeneous population (Lervåg, 2021).

### Research questions

We adopted a data‐driven approach to identify and understand the nature of children's reading comprehension difficulties, using data from the Avon Longitudinal Study of Parents and Children (ALSPAC), a UK birth cohort study that began in the early 1990s. Participants (*n* = 6846) were assessed at 8–9 years on measures of reading‐related skills: decoding, reading comprehension, and listening comprehension (hereafter referred to as “reading skills” for brevity). We first asked whether profiles of reading skills in this cohort reflect the Simple View of Reading. We used latent profile analysis (LPA) to extract different classes of readers without imposing arbitrary thresholds to better capture the dimensionality of reading difficulties. If reading comprehension is the product of variation in reading accuracy and language comprehension, we anticipated at least four classes to emerge, reflecting relative strengths and weaknesses in these domains. Any additional classes were expected to further differentiate by levels of ability (as in Torppa et al., 2007).

Using the poor comprehenders identified by the first analysis, we fitted a second model that incorporated additional cognitive and behavioural measures (reading rate, vocabulary, working memory, nonverbal ability, inattention; referred to collectively as “cognitive skills”) to test whether there are multiple distinct profiles within the group. We predicted that most would have language weaknesses, and that some would show additional weaknesses in nonverbal ability, working memory, and/or attention. We had no strong predictions over the extent to which these difficulties would co‐occur or reflect distinct cognitive profiles.

## ANALYSIS 1A (PREREGISTERED): IDENTIFYING READING PROFILES

### Method

#### Sample

The Avon Longitudinal Study of Parents and Children recruited 15,454 pregnancies in the former Avon area (UK) between April 1991 and December 1992, from whom 13,988 offspring were alive at one year. Later recruitment of eligible children at age 7 increased this total sample size to 14,901. The offspring have been studied ever since via a wide range of questionnaires and clinic assessments (Boyd et al., 2013; Fraser et al., 2013). The study website contains details of all the data that is available through a fully searchable data dictionary and variable search tool (http://www.bristol.ac.uk/alspac/researchers/our‐data/). Ethical approval for the study was obtained from the ALSPAC Ethics and Law Committee and the Local Research Ethics Committees. Informed consent for the use of data collected via questionnaires and clinics was obtained from participants following the recommendations of the ALSPAC Ethical and Law Committee at the time.

We report data from participants who completed the Neale Analysis of Reading Ability (NARA‐II; Neale, 1997) during a clinic visit at age 9.5 years (*n* = 6935). Approximately 61% of eligible participants attended the clinic, which was influenced by socio‐demographic factors: attendees were more likely to have mothers who were older, more highly educated, and homeowners, relative to eligible participants that did not attend. A higher proportion of eligible females versus males and white versus non‐white children attended the clinic. To address non‐independence, one twin from each pair (*n* = 89) was selected at random. Our final sample comprised 6846 children.

#### Measures

Reading assessments were administered during a clinic visit at age 9.5 years, with listening comprehension and all other cognitive assessments at age 8.5. We describe manual‐reported reliability for the full subtests for the relevant age. Analyses were based on raw scores, with age (months) included as a covariate unless otherwise stated. Summary statistics are presented in Table 1, alongside correlations between measures in Table 2.

**TABLE 1:** Summary statistics for all reading and cognitive variables (across whole analytic sample; n = 6846).

	% Missing	*M*	*SD*	min	max	skew
Item accuracy	0.57	12.72	4.63	0	20	−0.73
Passage accuracy	0	66.1	20.51	0	100	−0.49
Reading comprehension	0	24.99	7.84	0	44	−0.25
Listening comprehension	14.52	7.49	1.94	2	15	0.06
Vocabulary: Picture naming	14.87	7.47	1.83	0	10	−0.85
Vocabulary: Definitions	14.87	23.45	7.87	0	48	0.44
Reading rate	0.26	80.75	27.69	14	394	0.86
Nonverbal ability (Z‐composite)	14.93	0	0.62	−2.72	2.62	−0.2
Working memory	16.29	3.52	0.83	0	7	0.29
Inattention	15.1	2.9	2.24	0	10	0.83

**TABLE 2:** Correlations between all reading and cognitive variables.

	Item acc.	Passage acc.	Reading comp.	Listening comp.	Language: Picture naming	Language: Definitions	Reading rate	Nonverbal ability	Working memory	Inattention
Item accuracy	1	0.83	0.70	0.23	–	–	–	–	–	–
Passage accuracy	0.84	1	0.82	0.29	–	–	–	–	–	–
Reading comprehension	0.62	0.89	1	0.42	–	–	–	–	–	–
Listening comprehension	0.24	0.32	0.36	1	–	–	–	–	–	–
Vocabulary: Picture naming	0.35	0.44	0.47	0.39	1	–	–	–	–	–
Vocabulary: Definitions	0.33	0.45	0.49	0.38	0.45	1	–	–	–	–
Reading rate	0.50	0.59	0.55	0.25	0.29	0.30	1	–	–	–
Nonverbal ability	0.30	0.39	0.44	0.27	0.39	0.38	0.25	1	–	–
Working memory	0.32	0.38	0.34	0.19	0.27	0.20	0.20	0.33	1	–
Inattention	−0.25	−0.31	−0.32	−0.06	−0.12	−0.11	−0.16	−0.19	−0.17	1

*Note*: The top right quadrant reflects correlations between measures in Analysis 1, with the whole sample of 6846 participants. The bottom left quadrant reflects correlations between measures in Analysis 2, with the subsample of 947 weak comprehenders.

##### Decoding

**Item accuracy.** Children read ten “made‐up” and 10 real words aloud, selected from Nunes et al. (2003). The two lists have test‐retest reliabilities of 0.8 and 0.73 respectively. We intended to analyse words and nonwords as separate measures, but high word reading caused problems in higher‐class models. Data were therefore summed into a single score (/20).

**Passage accuracy.** In the NARA‐II children read aloud passages of increasing difficulty. They are instructed to attempt difficult words (and errors are corrected by the administrator), and to read carefully despite the time being recorded. The number of errors are deducted from a manual‐specified threshold value for each passage, and then summed to produce an overall score in line with standard scoring practices (Form 2; reliability = 0.87).

##### Comprehension

**Reading.** Children are told that they will be asked questions after reading each passage in the NARA‐II. Questions are asked orally by the administrator, and children are permitted to look back at the text when making their responses. A high proportion of the questions required inferences from the text (86%; see Bowyer‐Crane & Snowling, 2005, for a further analysis of question types). Responses were summed (reliability = 0.95).

**Listening.** This comprised alternate questions from the listening comprehension subtest of the Wechsler Objective Language Dimensions (WOLD; Rust, 1996). The tester read aloud paragraphs and children responded verbally to comprehension questions (/16; full subtest reliability = 0.84).

#### Analyses

Mixture models incorporate a categorical latent variable to identify subpopulations (latent classes or profiles) in the data. Analyses were conducted using Mplus v8.5 (Muthén & Muthén, 1998‐2017) and the MplusAutomation package in R (Hallquist & Wiley, 2018). Plans were preregistered (https://osf.io/4zahf) following the template by van den Akker et al. (2019), with deviations noted throughout. All models used robust estimation to account for any non‐normality in the data. Missingness was predicted by variables already included in the planned analyses (Appendix Table  S1, S1), and dealt with using full information maximum likelihood. Scripts and annotated outputs are available (https://osf.io/zvjw4/); direct links to the final model details are provided in Appendix S4. Note that our analyses do not account for clustering within schools, and while we do not have individual‐level data on the reading instruction that they received, the national curriculum at that time specified phonics instruction alongside other literacy activities.

##### Preregistered analyses

We used split‐half cross‐validation to develop the model on an “exploratory” half of the sample (*n* = 3423), with the confirmatory validation detailed below. An initial confirmatory factor analysis was used to inform our mixture model approach: we examined model fit for a single‐ versus two‐factor model (decoding, comprehension). The latter demonstrated that the correlation between factors was high (0.77), and listening comprehension was only weakly loaded (0.38) on the comprehension factor alongside reading comprehension. Thus, we proceeded to conduct a LPA directly using the observed measures (with age included as a covariate for each), rather than incorporate continuous latent factors for decoding and comprehension in a factor mixture model (FMM).

Following the class enumeration procedure set out by Masyn (2013), we fitted a series of models with increasing *k*‐classes under five model specifications that differed in whether the variances and/or covariances were estimated separately across classes (Masyn, 2013; Pastor et al., 2007). For each, candidate *k*‐class models were identified based on absolute fit, information heuristics (BIC, Consistent Akaike's Information Criterion, Approximate Weight of Evidence criterion), and (Vuong‐)Lo‐Mendell‐Rubin (LMR) tests. Approximate Bayes Factors and the approximate correct model probability were also calculated, but these tended to favour higher *k*‐class models regardless. Candidate models were further inspected for classification utility (class separation, average posterior class probability, theoretical interpretability), and a single best model per specification was identified. The best models from each specification were subsequently compared to determine the final model for cross‐validation. To avoid the naming fallacy inherent in latent class modelling, profiles must display a good degree of similarity to theoretically motivated subgroups, and models must cross‐validate sufficiently well to ensure that profiles are stable (Weller et al., 2020).

### Results

**FIGURE 1:**
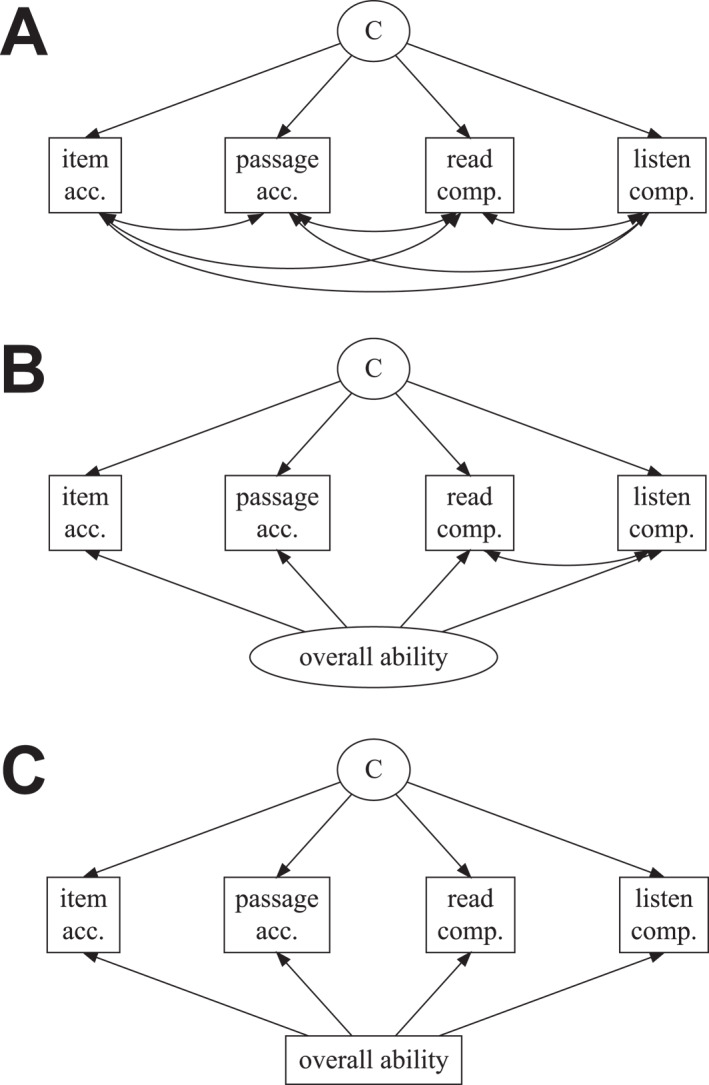
Final model specifications considered for identifying reading profiles (Analysis 1). All models incorporated age (months) as covariates for each task, not depicted. (A) Best‐fitting model specification from the preregistered LPAs, allowing separate variance and covariance estimation for each class; (B) Exploratory FMM that additionally estimated a continuous factor representing overall ability across tasks; (C) Exploratory LPA using overall ability score as a covariate on task performance.

Models that estimated covariances between measures showed better fit to the data than those that did not (Table S2). This specification also has theoretical support, given we incorporated multiple measures of decoding and comprehension that are expected to correlate. For each of these unrestricted specifications, models with 3‐5 classes emerged as the best candidates according to model fit and interpretability (Figure S1). The 4‐class model that allowed variances and covariances to vary between classes (Figure 1A) was selected as the best model with reasonable discrimination between classes. For brevity, only the *k*‐class models from this unrestricted model specification are presented in Table 3, allowing comparisons with the subsequent exploratory analyses.

**TABLE 3:** Fit indices for preregistered and exploratory mixture models used to identify reading profiles (Analysis 1).

Specification	*k*	*n* parameters	LL	BIC	CAIC	AWE	LMR *p*
Original LPA (Figure 1A)	1	23	−56151.3	112,489.7	112,512.7	112,524.2	‐
	2	40	−55212.9	110,751.3	110,791.3	110,811.3	<0.01
	3	57	−54753.1	109,970.0	110,027	110,055.5	<0.01
	**4**	**74**	**−54528.5**	**109,659.3**	**109,733.3**	**109,770.3**	**<0.01**
	5	91	−54325.8	109,392.2	109,483.2	109,528.7	0.01
	6	108	−54154.0	109,186.9	109,294.9	109,348.9	<0.01
	7	125	−54075.4	109,168.1	109,293.1	109,355.6	0.48
	8	142	−54036.3	109,228.3	109,370.3	109,441.3	–
Exploratory FMM with overall ability factor (Figure 1B)	1	21	−56156	112,482.8	112,503.8	112,514.3	–
	2	31	−55227.1	110,706.5	110,737.5	110,753.0	<0.01
	3	41	−54970.4	110,274.4	110,315.4	110,335.9	<0.01
	**4**	**51**	**−54766.8**	**109,948.6**	**109,999.6**	**110,025.1**	**<0.01**
	5	61	−54598.5	109,693.5	109,754.5	109,785	<0.01
	6	71	−54514.7	109,607.1	109,678.1	109,713.6	0.24
	7	81	−54426.9	109,512.9	109,593.9	109,634.4	0.51
	8	91	−54338.6	109,417.8	109,508.8	109,554.3	0.13
Exploratory LPA with overall ability covariate (Figure 1C)	1	21	−45149.8	90,470.58	90,491.58	90,502.08	‐
	2	32	−44243.1	88,746.56	88,778.56	88,794.56	<0.01
	3	43	−43389.6	87,129.12	87,172.12	87,193.62	<0.01
	4	54	−42901.6	86,242.6	86,296.6	86,323.6	0.22
	5	65	−42505.8	85,540.59	85,605.59	85,638.09	<0.01
	**6**	**76**	**−42129.9**	**84,878.21**	**84,954.21**	**84,992.21**	**<0.01**
	7	87	−41855.3	84,418.64	84,505.64	84,549.14	0.62
	8	98	−41593.7	83,984.84	84,082.84	84,131.84	0.2

*Note*: Rows in bold represent the best *k*‐class model selected for each model specification.

Abbreviations: *k*, number of classes; AWE, Approximate Weight of Evidence criterion; BIC, Bayesian Information Criterion; CAIC, Consistent Akaike's Information Criterion; LL, log likelihood; LMR, Lo‐Mendell‐Rubin.

The selected model showed low but acceptable entropy (0.70), with average latent class probabilities ranging from 0.78 to 0.89. The four classes showed differentiation by overall performance level across tasks, representing high ability (24.38%), high‐average ability (39.75%), and two lower ability classes (6.85% and 29.02%). This differentiation by level was common to all model specifications and candidate *k*‐class models considered.

### Interim summary

The preregistered analysis did not succeed in finding latent classes of readers. Our hypothesised profile of readers with good decoding but poor comprehension skills was not identified within the dataset. Rather, reading difficulties most commonly spanned both decoding and comprehension components, leaving only ordered profiles of ability. These profiles are of limited use beyond a continuous approach to modelling reading‐related skills and draw into question the view of reading difficulties as strongly categorical in nature. That is, based on the measures in our analysis, we did not find distinct groups of readers with isolated impairments in either decoding or comprehension.

An alternative approach emphasises this continuous variation in reading, and a number of studies have considered reading comprehension difficulties that are weak relative to decoding across the spectrum of reading ability (e.g., Tong et al., 2011; Wagner et al., 2021). Understanding the cognitive difficulties of children who show such uneven profiles of reading remains important, as are their implications for educational outcomes. Thus, to further inform discussions on conceptualising reading comprehension difficulties, we explored two additional model specifications designed to isolate qualitatively distinct profiles (i.e., profiles of varying strengths and weaknesses across tasks) in the context of strong differences in overall ability, as proposed by Morin and Marsh (2015).

## ANALYSIS 1B (EXPLORATORY): IDENTIFYING RELATIVE WEAKNESSES

### Method

We repeated the class enumeration sequence for two alternative model specifications. The first was a FMM that incorporated an additional continuous latent variable formed from all measures (Figure 1B), allowing for the estimation of qualitatively different profiles *alongside* differences in overall ability estimated by the continuous factor. The second was an LPA model that incorporated the higher‐order factor score as a control measure for each indicator variable (Figure 1C), allowing for the estimation of qualitatively different profiles *beyond* differences in overall ability. We selected the best candidate model from each specification (including from Analysis 1A), and then compared the model fit, stability, and theoretical relevance to select a final model from all specifications considered. The selected model was further scrutinised through cross‐validation in the second half of the sample.

### Results

The best‐fitting FMM with continuous latent variable had 4 classes, with good entropy (0.85) and average latent class probabilities of 0.85–0.94. This model also produced ordered profiles, namely a majority high‐average ability group (72.99%), two smaller classes of low‐average ability (6.08% and 4.48%), and a lower ability group (16.46%). However, the modified LPA with covariate did identify reading profiles with different shapes beyond overall ability in models above 5 classes, including a profile with relatively strong decoding and relatively weak comprehension that remained stable across 5‐6 class models. The main difference between these two models was the addition of a profile with relatively good comprehension. We favoured the 6‐class model as this profile conforms theoretically to previous research. This 6‐class solution had good entropy (0.80) and average latent class probabilities ranging from 0.82 to 0.90. The resultant profiles are described in more detail below.

Across all model specifications considered for Analysis 1A and 1B, the modified LPA with covariate clearly showed the best fit to the data (BIC = 84,878.21) compared to the alternative options (preregistered LPA: BIC = 109,659.26; FMM: BIC = 109,948.62; Table 3). Further, it was the only model that provided classes of theoretical and practical relevance to our goal of identifying individuals with relatively weak comprehension.

#### Final model

##### Cross‐validation

Full details are reported in Appendix S2. Our selected model showed poorer fit than a freely estimated model on the second half of the data (*p* < 0.001), largely driven by slight variations in class intercepts. The overall shape of the profiles remained similar. We repeated the class enumeration process for the second half of the sample. This confirmed that the 6‐class model showed the best profile stability across both halves of the sample. We describe these profiles below, re‐fitted to the full dataset.

##### Class labels

**FIGURE 2:**
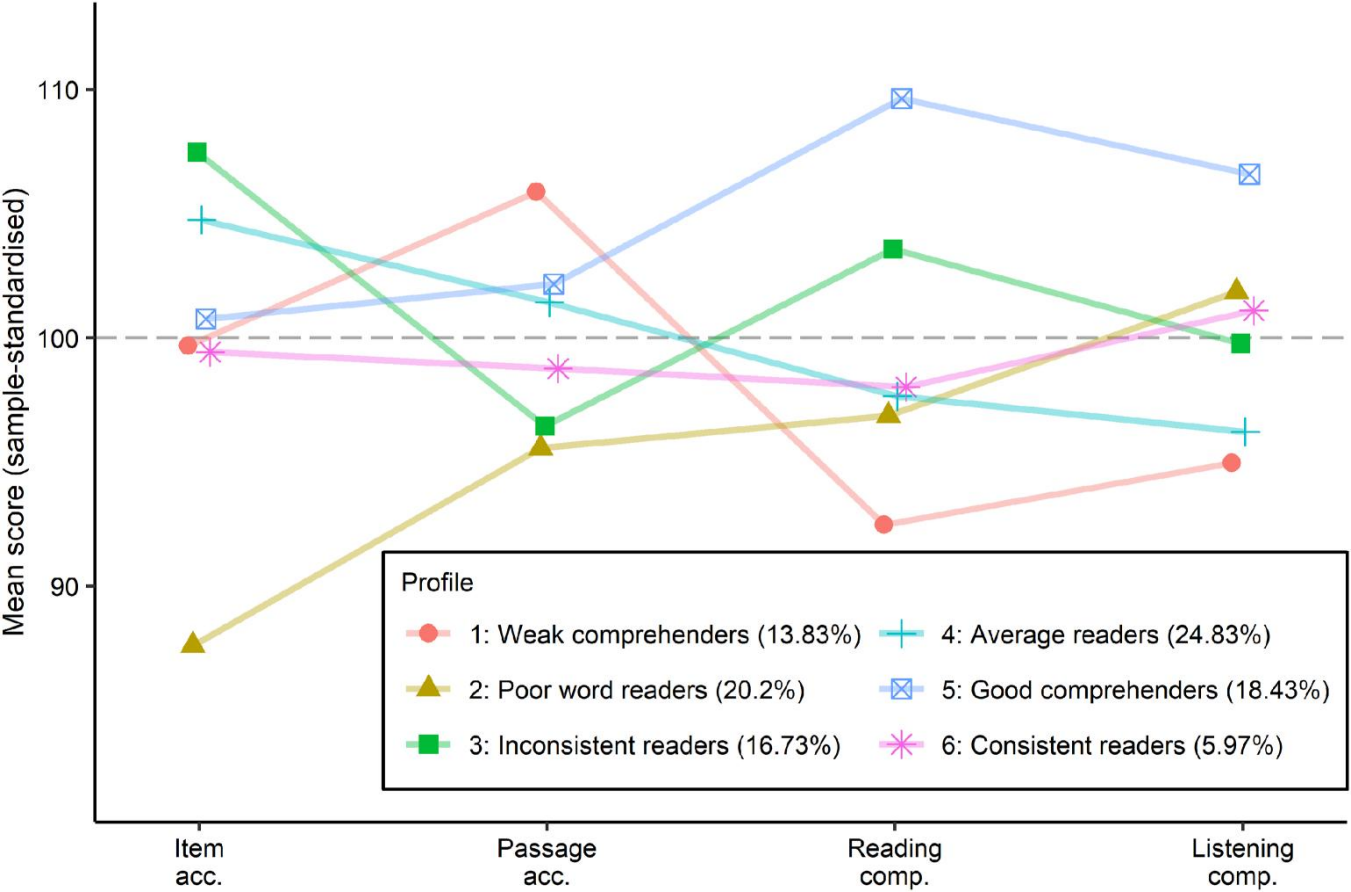
Mean performance on the reading skill measures for each reading profile (Analysis 1B). These reading profiles reflect those extracted from Analysis 1B: Exploratory LPA with overall ability covariate. Descriptive statistics are also provided in Table 4.

As summarised in Figure 2 and Table 4, Profile 1 (*weak comprehenders*; 13.83%) showed average‐good decoding, but were the lowest performers on comprehension. Profile 2 (*poor word readers*; 20.2%) had the opposite profile with weakest performance on item accuracy, low performance on passage accuracy, but relative strengths in listening comprehension. Profile 3 (*inconsistent readers*; 16.43%) had strengths in item accuracy and good‐average reading comprehension, but slightly weaker passage accuracy, perhaps reflecting differences in fluency given the timed nature of passage accuracy. Profile 4 (*average readers*; 24.83%) was largest with average‐good decoding and slightly weaker comprehension, but these differences were close to average and less extreme than in Profile 1. Profile 5 (*good comprehenders*; 18.43%) showed close‐to‐average decoding and were the highest performers on the two comprehension tasks. Profile 6 (*consistent readers*; 5.97%) performed close to average on all tasks. This small group was older at the time of the reading assessments (*M* = 123 vs. 118 months), and so the profile reflects superior performance in absolute terms but in line with age.

**TABLE 4:** Descriptive statistics of reading skills for each reading profile (Analysis 1B).

		Age (months) at 8.5 years clinic		Age (months) at 9.5 years clinic		Item accuracy		Passage accuracy		Reading comprehension		Listening comprehension	
Profile	n	M	(SD)	M	(SD)	M	(SD)	M	(SD)	M	(SD)	M	(SD)
Weak comprehenders	947	103.04	(1.94)	118.38	(3.45)	99.68	(15.63)	105.87	(14.24)	92.47	(12.43)	94.96	(14.09)
Poor word readers	1383	102.88	(1.94)	118.22	(3.42)	87.61	(15.40)	95.56	(17.27)	96.86	(16.19)	101.85	(14.61)
Inconsistent readers	1145	102.94	(2.03)	118.22	(3.73)	107.47	(9.15)	96.45	(10.80)	103.57	(13.06)	99.78	(14.05)
Average readers	1700	102.84	(1.92)	118.41	(3.55)	104.75	(12.30)	101.42	(14.15)	97.65	(12.76)	96.21	(13.84)
Good comprehenders	1262	102.85	(1.91)	118.59	(3.73)	100.76	(12.73)	102.17	(14.53)	109.64	(13.92)	106.58	(15.20)
Consistent readers	409	115.08	(3.55)	123.51	(6.1)	99.43	(15.93)	98.77	(16.55)	98.02	(16.07)	101.1	(15.91)

*Note*: These reading profiles reflect those extracted from Analysis 1B: Exploratory LPA with overall ability covariate. Assessment scores reflect scores standardised on the present sample (*n* = 6846).

Given that the weak comprehender group is of primary interest for Analysis 2, Table 5 presents the Cohen's *d* effect sizes for this group relative to each other profile, on all reading measures.

**TABLE 5:** Effect sizes for differences in reading skills between weak comprehenders and each of the five other reading profiles (Analysis 1B).

	2: Poor word readers	3: Inconsistent readers	4: Average readers	5: Good comprehenders	6: Consistent readers
Age (months) at 8.5 years clinic	0.08	0.05	0.10	0.10	−4.77
Age (months) at 9.5 years clinic	0.05	0.04	−0.01	−0.06	−1.16
Item accuracy	0.78	−0.62	−0.37	−0.08	0.02
Passage accuracy	0.64	0.76	0.31	0.26	0.47
Reading comprehension	−0.30	−0.87	−0.41	−1.29	−0.41
Listening comprehension	−0.48	−0.34	−0.09	−0.79	−0.42

*Note*: These reading profiles reflect those extracted from Analysis 1B: Exploratory LPA with continuous covariate. Effect sizes reflect Cohen's *d* for group differences.

### Interim summary

Our initial models in Analysis 1A produced ordered profiles indicating strong differences in performance spanning decoding and comprehension. Controlling for overall ability in Analysis 1B, however, revealed qualitatively different reading profiles with varied strengths and weaknesses, including a group of individuals with weak reading comprehension relative to their decoding ability. This “weak comprehender” group differs from the traditionally identified poor comprehenders in the sense that not all individuals would be considered impaired below a threshold score, but aligns more closely with the “unexpected” poor comprehenders selected by regression approaches (e.g., MacKay et al., 2017; Tong et al., 2011). To better understand how this group aligns with prior research, and consider potential heterogeneity, we next examined whether there are multiple cognitively distinct profiles within this group who have weak comprehension relative to their decoding skills.

## ANALYSIS 2: COGNITIVE PROFILES OF WEAK COMPREHENDERS

### Method

#### Sample

This comprised the weak comprehender group identified in Analysis 1B (*n* = 947).

#### Measures

Adding to the reading measures in Analysis 1, we included the following measures from the same clinic visits. Distributional statistics and missingness are detailed in Table 1.

##### Language

**Vocabulary: Picture naming.** Children named a subset of 10 pictures (WOLD expressive vocabulary task; Rust, 1996). Correct answers were summed (/10). The split‐half reliability for the full oral expression subtest is 0.91.

**Vocabulary: Definitions.** Alternate items were administered from the vocabulary subtest, Weschler Intelligence Scale for Children (WISC‐III; Wechsler et al., 1992). Children named depicted objects in early items; later items required word definitions to be supplied (scored 0–2). Scores were summed and doubled (for comparability to the full test; reliability = 0.88).

We intended to incorporate separate variables for comprehension and vocabulary. As the two constructs were highly correlated, they were collapsed into a single latent factor labelled language.

##### Reading rate

The NARA‐II measures rate based on the average number of words in connected text read/minute (reliability across forms = 0.71). While reading rate is often considered a marker of decoding efficiency (particularly in more transparent orthographies than English), no studies to our knowledge have used the NARA‐II rate score to select poor comprehenders and therefore it was not included in Analysis 1. It is a multifaceted measure with many potential influencing factors (e.g., decoding errors, attention, articulation, anxiety), and thus we included it in Analysis 2 to explore its utility as a co‐occurring marker of different types of reading comprehension difficulty.

##### Nonverbal ability

We planned to include five WISC‐III Performance Intelligence Quotient (IQ) subtests (Picture Completion, Coding, Picture Arrangement, Block Design, Object Assembly) to indicate nonverbal ability, but these were collapsed to a single score to reduce model complexity. Reliability for the full Performance IQ scale is 0.90. Each subtest was z‐scored on the sample, and an averaged composite z‐score returned.

##### Working memory

In the WISC‐III Backward Digit Span, children repeat back increasingly long lists of digits in the reverse order. The number of digit strings reported correctly was treated as categorical and extreme values collapsed to form four ordered categories. Split‐half reliability is 0.84 for the full Digit Span subtest (forward and backward).

##### Inattention

The Strengths and Difficulties Questionnaire hyperactivity subscale (Goodman, 1997), was completed by parents when children were age 9. Goodman (2001) reports a Cronbach's α of 0.77 for this subscale. As this scale is discrete and highly skewed (∼50% of children scoring 0–2/10) it was analysed as categorical, with the highest two scores collapsed to meet the software constraint of 10 categories. No age covariate was included.

#### Analyses

##### Excluded measures

The structure of the final model deviates from that specified in the preregistration (https://osf.io/4zahf), as detailed in Appendix S3. Of note, we were unable to incorporate both Backward Digit Span and Counting Span working memory measures due to the resultant number of empty cells in each class; we favoured Digit Span as it was administered at the same time as the other variables. Three cognitive measures of attention were also excluded, due to poor distributions and poor loadings regardless of factor structure.

##### Factor mixture model

**FIGURE 3:**
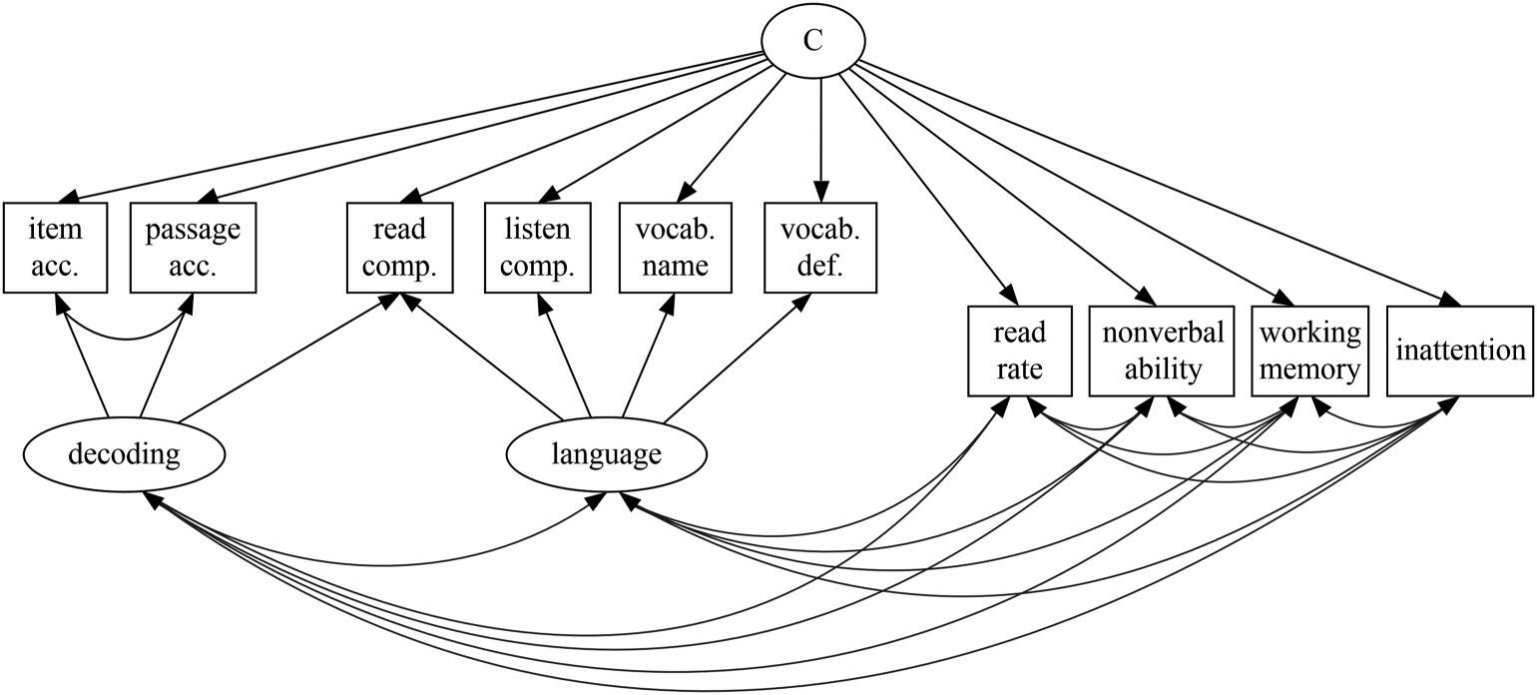
Final factor mixture model (FMM) specification for identifying the cognitive profiles of weak comprehenders (Analysis 2). Age (months) was incorporated as a covariate for all observed variables, except inattention. The four left‐most observed variables were also included as measures of reading skill in Analysis 1.

Figure 3 shows the final model specification. We followed the same class enumeration procedure as above for two model specifications that differed in whether factor loadings varied between classes (akin to models 3 and 4 from Clark et al., 2013). However, models with class‐specific factor loadings were too complex for the data, leading to errors in parameter estimation that could not be resolved without substantial simplification. We thus focussed on the model with constrained factor loadings across classes, allowing variances and covariances to be estimated separately for each class. As above, missingness was random conditional on variables already included in the model, and was dealt with using full information maximum likelihood (see Appendix S1, Table S1).

We inspected model fit using AIC, BIC, aBIC and adjusted LRTs, and considered the utility of classification in deciding on the best *k*‐class model. The computational load was deemed too high to compute the planned bootstrapped estimates for standard errors, but the large sample and the clear distinction between profiles gives confidence that any adjustments would not affect interpretation of the results.

### Results

Only the 2‐ and 3‐class models were supported by the data. Adding a fourth class resulted in an overly complex model for successful fit. The 3‐class model (Log Likelihood [LL] = −19621.32, AIC = 39,484.64, BIC = 40,071.89, aBIC = 39,687.60) showed slightly better fit than the 2‐class model (LL = 19,781.25, AIC = 39,738.50, BIC = 40,165.60, aBIC = 39,886.11), but this difference was not significant according to the (V)LMR tests (*p* = 0.12). Further, adding a third class introduced only a small profile (3.93%) that was not theoretically meaningful. The 2‐class model was therefore selected as most parsimonious.

**FIGURE 4:**
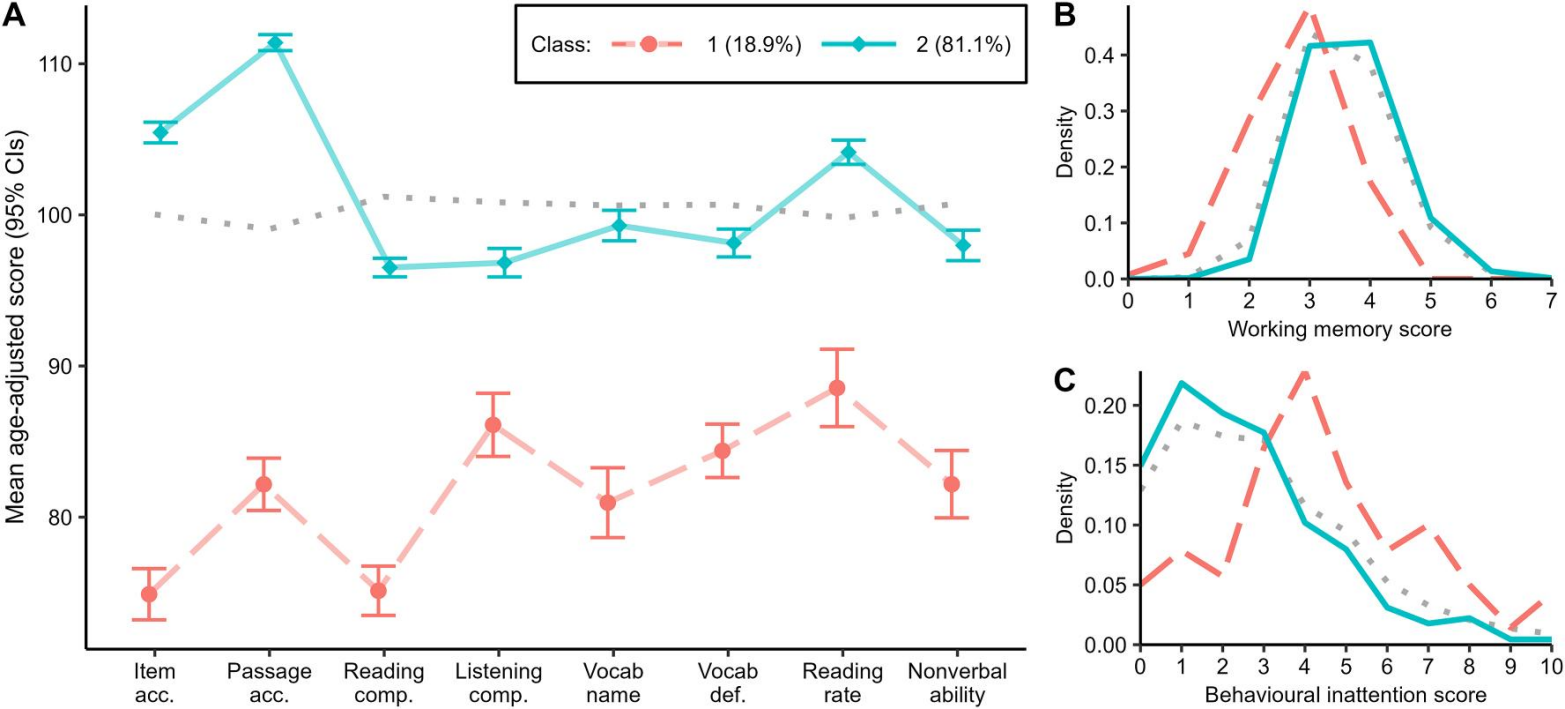
Descriptive statistics of reading and cognitive skills for each cognitive profile of weak comprehenders (Analysis 2). Assessment performance for Class 1 (Low ability) and 2 (Unexpected weak comprehenders); (A) Sample‐standardised scores of reading and cognitive skills (error bars denote 95% confidence intervals; the four left‐most measures were also included as measures of reading skill in Analysis 1); (B) distribution of working memory scores; and (C) distribution of inattention scores. For reference, the remainder of the sample (i.e., those not identified as weak comprehenders; n = 5899) are marked by the grey dotted line.

The 2‐class model was a significantly better fit than a 1‐class model (*p* = 0.01). It showed good entropy (0.88) and classification probabilities of 0.90 and 0.98. The two classes were dissociated across all measures. The smaller class (*low ability group*; 18.9% of poor comprehenders) showed relatively severe impairments across the board (Figure 4A & B), alongside increased hyperactivity/inattention (Figure 4C). Although their difficulties were most severe for reading comprehension and item accuracy (see also Figure S2), performance was close to or below ‐1*SD* age‐expected levels across the board.

The remaining 81.1% of poor comprehenders (*unexpected weak comprehenders*) showed largely typical performance across tasks: that is, although comprehension was substantially below decoding, performance across both domains tended to fall within average range. Decoding and rate were above‐average on age‐standardised scores; comprehension, vocabulary (definitions), and nonverbal ability were below‐average (Figure 4A). This group showed comparable—if not slightly superior—working memory and attention to the remainder of the sample not classed as weak comprehenders (*n* = 5899; Figure 4B & C). Effect sizes are presented in Table 6.

**TABLE 6:** Effect sizes for differences in reading and cognitive skills between the two cognitive profiles of weak comprehenders (Low ability vs. Unexpected; Analysis 2) and compared to the remaining sample (“Other”).

	Low ability versus Unexpected	Low ability versus Other	Unexpected versus Other
Age (months) at 8.5 years clinic	0.18	−0.1	−0.2
Age (months) at 9.5 years clinic	0.17	0.03	−0.11
Item accuracy	−3.03	−1.7	0.38
Passage accuracy	−3.45	−1.14	0.87
Reading comprehension	−2.33	−1.75	−0.32
Listening comprehension	−0.8	−0.99	−0.27
Vocabulary: Picture naming	−1.26	−1.33	−0.09
Vocabulary: Definitions	−1.07	−1.09	−0.17
Reading rate	−1.23	−0.74	0.29
Nonverbal ability	−1.1	−1.26	−0.19

*Note*: The Low Ability and Unexpected weak comprehender groups reflect the cognitive profiles extracted from Analysis 2. The “Other” group reflects the remainder of the sample (i.e., those not identified as weak comprehenders in Analysis 1; *n* = 5899), presented for reference. Effect sizes are computed as Cohen's *d*, for continuous variables only.

### Discussion

Using a data‐driven approach, our first analysis demonstrated that the traditional poor comprehender profile—as defined by reading comprehension impairments that are substantially below average‐good decoding skills—is not reflected in the data. However, it is possible to examine unexpectedly weak comprehension relative to decoding skills, adopting a dimensional approach to reading weaknesses. By accounting for general reading ability in an exploratory model, we were able to identify 947 children with weak comprehension relative to decoding. Focussing on this subgroup, comprehension difficulties were most consistently accompanied by weaknesses in oral language and nonverbal ability. More severe comprehension problems were associated with the additional presence of broader cognitive difficulties.

#### Dimensionality of reading difficulties

The traditional poor comprehender profile described in experimental literature was not well‐supported by the data: decoding and comprehension were highly correlated, and our initial LPA extracted profiles that varied only in overall ability. It is important to note that the absence of qualitatively different profiles in this sample is likely exacerbated by the measures available: the decoding and comprehension measures from the NARA‐II are highly correlated (0.82), and are also the measures with the most variance. Thus, it remains plausible that the hypothesised profiles would have emerged if entirely separate assessments of decoding and comprehension were used. However, these component skills are highly correlated in developing readers of this age (see García & Cain, 2014, for a meta‐analysis), and a recent study of Finnish readers also failed to identify discrepant profiles *despite* using measures from different tasks (Psyridou et al., 2021). These results contrast those of Torppa et al. (2007), who observed a poor comprehender group in a different sample of Finnish readers during the first two years of schooling (aged 7–9 years). Although the evidence is mixed (Psyridou et al., 2021), we tentatively consider that literacy development may also be important to understanding these conflicting findings. Foorman et al. (2017) found evidence of qualitatively distinct literacy profiles in early schooling, yet only ordered profiles emerged beyond 5^th^ grade (aged 10–11 years). This latter group better aligns with our sample, who were also in their fourth or fifth year of formal schooling when they completed the reading assessments. Thus, an important conclusion from the present study is that, at least later in development, reading difficulties are not strongly categorical and most commonly span problems with both decoding and comprehension.

Including overall reading ability as a covariate, we could extract qualitatively distinct profiles in the context of strong quantitative differences in overall ability. Thus, children with *relatively* weak comprehension compared to decoding were identified in a data‐driven way, in line with the view that reading comprehension difficulties are dimensional in nature (Wagner et al., 2021). This approach is somewhat similar to regression‐based analyses that identify children whose comprehension is weaker than predicted by age and decoding (e.g., Tong et al., 2011). However, our data‐driven approach does not require arbitrary decisions (e.g., the size of the comprehension gap). Inspection of the alternative models considered for identifying reading profiles indicated that this approach was the best fit in accounting for the data, and cross‐validated well across different halves of the sample. Whether these profiles of uneven reading skills make useful predictions about children's outcomes beyond the severity of overall reading difficulties is a key question for future research.

#### Cognitive profile(s) of weak comprehenders

Consistent with previous research that has documented oral language weaknesses in poor comprehenders (e.g., Nation et al., 2004), the weak comprehenders (*n* = 947) selected by our dimensional approach had weak vocabulary, particularly for definitions, a task that taps depth of lexical‐semantic knowledge. They also showed lower nonverbal ability, reinforcing the view that diagnostic criteria for reading and language disorders should not include average‐range nonverbal ability (e.g., Norbury et al., 2016). This is further supported by evidence of mutualistic influences between verbal and nonverbal abilities across development (Kievit et al., 2019), indicating that weaknesses tightly restricted to the language domain are unlikely to be observed in later childhood.

We also investigated whether there were distinct “subtypes” within the weak comprehender group that might indicate distinct causes of reading comprehension difficulty. The data did not support qualitatively different profiles with diverse areas of weakness. Instead, severity might depend on the accumulation of cognitive strengths and weaknesses. The majority of weak comprehenders showed typical working memory, in line with previous findings (Pimperton & Nation, 2014). Hyperactivity levels were also low—lower even than the remaining sample without weak comprehension. Importantly however, a subset of weak comprehenders showed poor performance across domains, and notably, also the most severe comprehension impairments. These observations align with a multiple deficit view that sees risk for reading disorder on a continuum, with co‐occurring strengths and weaknesses in other cognitive domains influencing severity (Snowling, 2008). Indeed, Hayiou‐Thomas et al. (2021) found that the breadth rather than the severity of risk factors best predicts reading and language outcomes.

#### Implications for the classroom, limitations, and future directions

The influence of the Simple View of Reading (Hoover & Gough, 1990) has been building in educational policy and practice (e.g., Rose, 2006; for discussion, see Vaughn, 2018). This is to be welcomed as it captures the complexity and multifaceted nature of reading, while emphasising decoding and comprehension as the core elements of successful reading. It provides a framework to help understand variation in reading comprehension and in turn, this offers guiding principles for assessment and intervention. Our findings sit comfortably within this framework, but they also caution against a strong categorical perspective and instead demonstrate that reading comprehension difficulties are best considered along a continuum *relative* to, rather than in contrast with, decoding skills. This conclusion aligns with recent discussions in the context of developmental dyslexia. Dyslexia is traditionally associated with specific decoding difficulties that are underpinned by phonological weaknesses, and so might be considered the mirror profile of poor comprehenders, traditionally defined. Yet, many children with dyslexia have broader language and cognitive weaknesses that can be considered alongside poor decoding as variation along multiple continua that confer risk for poor reading comprehension (e.g., Snowling & Hulme, 2021). What follows from this is that some dyslexic children may benefit from more than just targeted decoding support. Likewise for the children identified as weak comprehenders in our analyses. The majority showed poor reading comprehension relative to decoding, and weaknesses in vocabulary and nonverbal ability. In addition, accompanying strengths and weaknesses in other cognitive domains influenced the severity of comprehension outcomes, and those with the most severe difficulties with reading comprehension showed broader difficulties across domains. Thus, while oral language is a key target for intervention (e.g., Clarke et al., 2010), practitioners should look beyond language to identify broader strengths and weaknesses that might contribute to reading comprehension outcomes. More generally, the Simple View of Reading captures this complexity on the basis that the two core elements of reading comprehension (decoding and language comprehension) are themselves complex and multifaceted. It is important that this complexity is not overlooked when using the Simple View to guide classroom practice (e.g., Castles et al., 2018; Catts, 2018; Snow, 2018).

The profiles we extracted were limited by the measures available. In particular, the decoding factor was not well‐identified, and the availability of more diverse reading and cognitive assessments would have permitted a more thorough exploration of different factor structures that better align with alternative models of reading (as in Foorman et al., 2015, for example). The availability of broader measures might also permit the estimation of different measurement parameters across classes, allowing for the consideration of how strengths and weaknesses may interact differently across development for different groups of children (such a model was too complex for the current dataset). As discussed above, our findings do not exclude the possibility that distinct profiles exist earlier in development, indicative of distinct causes, or that mutualistic influences in cognitive development lead to broader weaknesses by mid‐childhood. Our findings highlight the importance of using data‐driven longitudinal approaches to address these questions in future.

## AUTHOR CONTRIBUTION

**Emma James:** Conceptualization, Data curation, Formal analysis, Funding acquisition, Investigation, Methodology, Project administration, Visualization, Writing – original draft, Writing – review & editing. **Paul Thompson:** Conceptualization, Formal analysis, Funding acquisition, Methodology, Validation, Writing – review & editing. **Lucy Bowes:** Conceptualization, Funding acquisition, Methodology, Writing – review & editing. **Kate Nation:** Conceptualization, Funding acquisition, Methodology, Supervision, Writing – review & editing.

## CONFLICT OF INTEREST STATEMENT

The authors have declared that they have no competing or potential conflicts of interest.

### OPEN RESEARCH BADGES

This article has earned a Preregistered Research Designs badge for having a preregistered research design, available at https://osf.io/4zahf.

## ETHICAL CONSIDERATIONS

Ethical approval for the study was obtained from the ALSPAC Ethics and Law Committee and the Local Research Ethics Committees. Informed consent for the use of data collected via questionnaires and clinics was obtained from participants following the recommendations of the ALSPAC Ethical and Law Committee at the time.

## Supporting information

Supplementary Material

## Data Availability

The informed consent obtained from ALSPAC participants does not allow the data to be made freely available through any third party maintained public repository. However, data used for this submission can be made available on request to the ALSPAC Executive. The study website contains details of all the data that is available through a fully searchable data dictionary and variable search tool (http://www.bristol.ac.uk/alspac/researchers/our‐data/). Annotated output files from our analyses are available at https://osf.io/zvjw4/.

## References

[jcv212177-bib-0001] Astle, D. E. , & Fletcher‐Watson, S. (2020). Beyond the core‐deficit hypothesis in developmental disorders. Current Directions in Psychological Science, 29(5), 431–437. 10.1177/0963721420925518 33071483 PMC7539596

[jcv212177-bib-0002] Bowyer‐Crane, C. , & Snowling, M. J. (2005). Assessing children’s inference generation: What do tests of reading comprehension measure? British Journal of Educational Psychology, 75(2), 189–201. 10.1348/000709904X22674 16033662

[jcv212177-bib-0003] Boyd, A. , Golding, J. , Macleod, J. , Lawlor, D. A. , Fraser, A. , Henderson, J. , Molloy, L. , Ness, A. , Ring, S. , & Davey Smith, G. (2013). Cohort profile: The ‘children of the 90s’—The index offspring of the Avon longitudinal study of parents and children. International Journal of Epidemiology, 42(1), 111–127. 10.1093/ije/dys064 22507743 PMC3600618

[jcv212177-bib-0004] Cain, K. , & Bignell, S. (2014). Reading and listening comprehension and their relation to inattention and hyperactivity. British Journal of Educational Psychology, 84(1), 108–124. 10.1111/bjep.12009 24547756

[jcv212177-bib-0005] Cain, K. , & Oakhill, J. (2006). Profiles of children with specific reading comprehension difficulties. British Journal of Educational Psychology, 76(4), 683–696. 10.1348/000709905X67610 17094880

[jcv212177-bib-0006] Carretti, B. , Borella, E. , Cornoldi, C. , & Beni, R. D. (2009). Role of working memory in explaining the performance of individuals with specific reading comprehension difficulties: A meta‐analysis. Learning and Individual Differences, 6(2), 246–251. 10.1016/j.lindif.2008.10.002

[jcv212177-bib-0007] Castles, A. , Rastle, K. , & Nation, K. (2018). Ending the reading wars: Reading acquisition from novice to expert. Psychological Science in the Public Interest, 19(1), 5–51. 10.1177/1529100618772271 29890888

[jcv212177-bib-0008] Catts, H. W. (2018). The simple view of reading: Advancements and false impressions. Remedial and Special Education, 39(5), 317–323. 10.1177/0741932518767563 PMC6530938 31130774

[jcv212177-bib-0009] Catts, H. W. , Adlof, S. , & Ellis Weismer, S. (2006). Language deficits In poor comprehenders: A case for the simple view of reading. Journal of Speech, Language, and Hearing Research : JSLHR, 49(2), 278–293. 10.1044/1092-4388(2006/023 16671844

[jcv212177-bib-0010] Clark, S. L. , Muthén, B. , Kaprio, J. , D’Onofrio, B. M. , Viken, R. , & Rose, R. J. (2013). Models and strategies for factor mixture analysis: An example concerning the structure underlying ps. Structural Equation Modeling: A Multidisciplinary Journal, 20(4), 681–703. 10.1080/10705511.2013.824786 PMC3844130 24302849

[jcv212177-bib-0011] Clarke, P. J. , Snowling, M. J. , Truelove, E. , & Hulme, C. (2010). Ameliorating children’s reading‐comprehension difficulties: A randomized controlled trial. Psychological Science, 21(8), 1106–1116. 10.1177/0956797610375449 20585051

[jcv212177-bib-0012] Colenbrander, D. , Kohnen, S. , Smith‐Lock, K. , & Nickels, L. (2016). Individual differences in the vocabulary skills of children with poor reading comprehension. Learning and Individual Differences, 50, 210–220. 10.1016/j.lindif.2016.07.021

[jcv212177-bib-0013] Elwér, Å. , Gustafson, S. , Byrne, B. , Olson, R. K. , Keenan, J. M. , & Samuelsson, S. (2015). A retrospective longitudinal study of cognitive and language skills in poor reading comprehension. Scandinavian Journal of Psychology, 56(2), 157–166. 10.1111/sjop.12188 25581078 PMC4356634

[jcv212177-bib-0014] Foorman, B. R. , Koon, S. , Petscher, Y. , Mitchell, A. , & Truckenmiller, A. (2015). Examining general and specific factors in the dimensionality of oral language and reading in 4th–10th grades. Journal of Educational Psychology, 107(3), 884–899. 10.1037/edu0000026 26346839 PMC4557887

[jcv212177-bib-0015] Foorman, B. R. , Petscher, Y. , Stanley, C. , & Truckenmiller, A. (2017). Latent profiles of reading and language and their association with standardized reading outcomes in kindergarten through tenth grade. Journal of Research on Educational Effectiveness, 10(3), 619–645. 10.1080/19345747.2016.1237597 30918534 PMC6433153

[jcv212177-bib-0016] Fraser, A. , Macdonald‐Wallis, C. , Tilling, K. , Boyd, A. , Golding, J. , Smith, G. D. , Henderson, J. , Macleod, J. , Molloy, L. , Ness, A. , Ring, S. , Nelson, S. M. , & Lawlor, D. A. (2013). Cohort profile: The Avon longitudinal study of parents and children: ALSPAC Mothers cohort. International Journal of Epidemiology, 42(1), 97–110. 10.1093/ije/dys066 22507742 PMC3600619

[jcv212177-bib-0017] García, J. R. , & Cain, K. (2014). Decoding and reading comprehension: A meta‐analysis to identify which reader and assessment characteristics influence the strength of the relationship in English. Review of Educational Research, 84(1), 74–111. 10.3102/0034654313499616

[jcv212177-bib-0018] Goodman, R. (1997). The strengths and difficulties questionnaire: A research note. Journal of Child Psychology and Psychiatry, 38(5), 581–586. 10.1111/j.1469-7610.1997.tb01545.x 9255702

[jcv212177-bib-0019] Goodman, R. (2001). Psychometric properties of the strengths and difficulties questionnaire. Journal of the American Academy of Child & Adolescent Psychiatry, 40(11), 1337–1345. 10.1097/00004583-200111000-00015 11699809

[jcv212177-bib-0020] Hallquist, M. N. , & Wiley, J. F. (2018). MplusAutomation: An R package for facilitating large‐scale latent variable analyses in Mplus. Structural Equation Modeling: A Multidisciplinary Journal, 25(4), 621–638. 10.1080/10705511.2017.1402334 30083048 PMC6075832

[jcv212177-bib-0021] Hayiou‐Thomas, M. E. , Smith‐Woolley, E. , & Dale, P. S. (2021). Breadth versus depth: Cumulative risk model and continuous measure prediction of poor language and reading outcomes at 12. Developmental Science, 24(1), e12998. 10.1111/desc.12998 32449284 PMC11475567

[jcv212177-bib-0022] Hoover, A. , & Gough, B. (1990). The simple view of reading. Reading and Writing, 2, 127–160. 10.1007/BF00401799

[jcv212177-bib-0023] Justice, L. , Mashburn, A. , & Petscher, Y. (2013). Very early language skills of fifth‐grade poor comprehenders. Journal of Research in Reading, 36(2), 172–185. 10.1111/j.1467-9817.2011.01498.x 25620819 PMC4301613

[jcv212177-bib-0024] Kievit, R. A. (2020). Sensitive periods in cognitive development: A mutualistic perspective. Current Opinion in Behavioral Sciences, 36, 144–149. 10.1016/j.cobeha.2020.10.007

[jcv212177-bib-0025] Kievit, R. A. , Hofman, A. D. , & Nation, K. (2019). Mutualistic Coupling Between Vocabulary and Reasoning in Young Children: A Replication and Extension of the Study by Kievit et al. (2017). Psychological Science, 30(8), 1245–1252. 10.1177/0956797619841265 31100049 PMC6691592

[jcv212177-bib-0026] Landi, N. , & Ryherd, K. (2017). Understanding specific reading comprehension deficit: A review. Language and Linguistics Compass, 11(2), e12234. 10.1111/lnc3.12234 30034511 PMC6051548

[jcv212177-bib-0027] Lervåg, A. (2021). Editorial: Is there a core deficit in specific learning disabilities? Journal of Child Psychology and Psychiatry, 62(6), 677–679. 10.1111/jcpp.13434 34008198

[jcv212177-bib-0028] Logan, J. , & Logan, J. (2017). Pressure points in reading comprehension: A quantile multiple regression analysis. Journal of Educational Psychology, 109(4), 451–464. 10.1037/edu0000150

[jcv212177-bib-0029] MacKay, E. J. , Levesque, K. , & Deacon, S. H. (2017). Unexpected poor comprehenders: An investigation of multiple aspects of morphological awareness. Journal of Research in Reading, 40(2), 125–138. 10.1111/1467-9817.12108

[jcv212177-bib-0030] Masyn, K. E. (2013). 25 latent class analysis and finite mixture modeling. The Oxford Handbook of Quantitative Methods, 551.

[jcv212177-bib-0031] Melby‐Lervåg, M. , & Lervåg, A. (2014). Effects of educational interventions targeting reading comprehension and underlying components. Child Development Perspectives, 8(2), 96–100. 10.1111/cdep.12068

[jcv212177-bib-0032] Morin, A. J. S. , & Marsh, H. W. (2015). Disentangling shape from level effects in person‐centered analyses: An illustration based on University teachers’ multidimensional profiles of effectiveness. Structural Equation Modeling: A Multidisciplinary Journal, 22(1), 39–59. 10.1080/10705511.2014.919825

[jcv212177-bib-0033] Muthén, L. K. , & Muthén, B. O. (1998). Mplus user’s guide (eighth). Muthén & Muthén.

[jcv212177-bib-0034] Nation, K. , Clarke, P. , Marshall, C. M. , & Durand, M. (2004). Hidden Language Impairments in children. Journal of Speech, Language, and Hearing Research, 47(1), 199–211. 10.1044/1092-4388(2004/017 15072539

[jcv212177-bib-0035] Nation, K. , Cocksey, J. , Taylor, J. S. H. , & Bishop, D. V. M. (2010). A longitudinal investigation of early reading and language skills in children with poor reading comprehension. Journal of Child Psychology and Psychiatry, 51(9), 1031–1039. 10.1111/j.1469-7610.2010.02254.x 20456536

[jcv212177-bib-0036] Neale, M. D. (1997). Neale analysis of reading ability—second revised British edition. NFER‐Nelson.

[jcv212177-bib-0037] Norbury, C. F. , Gooch, D. , Wray, C. , Baird, G. , Charman, T. , Simonoff, E. , Vamvakas, G. , & Pickles, A. (2016). The impact of nonverbal ability on prevalence and clinical presentation of language disorder: Evidence from a population study. Journal of Child Psychology and Psychiatry, 57(11), 1247–1257. 10.1111/jcpp.12573 27184709 PMC5082564

[jcv212177-bib-0038] Nunes, T. , Bryant, P. , & Olsson, J. (2003). Learning morphological and phonological spelling rules: An intervention study. Scientific Studies of Reading, 7(3), 289–307. 10.1207/S1532799XSSR0703_6

[jcv212177-bib-0039] Pastor, D. A. , Barron, K. E. , Miller, B. J. , & Davis, S. L. (2007). A latent profile analysis of college students’ achievement goal orientation. Contemporary Educational Psychology, 32(1), 8–47. 10.1016/j.cedpsych.2006.10.003

[jcv212177-bib-0040] Pennington, B. F. (2006). From single to multiple deficit models of developmental disorders. Cognition, 101(2), 385–413. 10.1016/j.cognition.2006.04.008 16844106

[jcv212177-bib-0041] Perfetti, C. , Stafura, J. , & Adlof, S. (2014). Reading comprehension and reading comprehension problems: A word‐to‐text integration perspective. In Unravelling reading comprehension: Behavioral, neurobiological, and genetic components (pp. 22–32).

[jcv212177-bib-0042] Pimperton, H. , & Nation, K. (2010). Suppressing irrelevant information from working memory: Evidence for domain‐specific deficits in poor comprehenders. Journal of Memory and Language, 12(4), 380–391. 10.1016/j.jml.2010.02.005

[jcv212177-bib-0043] Pimperton, H. , & Nation, K. (2014). Poor comprehenders in the classroom: Teacher ratings of behavior in children with poor reading comprehension and its relationship with individual differences in working memory. Journal of Learning Disabilities, 47(3), 199–207. 10.1177/0022219412454172 22907886

[jcv212177-bib-0044] Psyridou, M. , Tolvanen, A. , de Jong, P. F. , Lerkkanen, M.‐K. , Poikkeus, A.‐M. , & Torppa, M. (2021). Developmental profiles of reading fluency and reading comprehension from grades 1 to 9 and their early identification. Developmental Psychology, 57(11), 1840–1854. 10.1037/dev0000976 34914449

[jcv212177-bib-0045] Rose, J. (2006). Independent review of the teaching of early reading (Department for Education and Skills).

[jcv212177-bib-0046] Rust, J. (1996). Wechsler Objective Language Dimensions manual. The Psychological Corporation.

[jcv212177-bib-0047] Snow, C. E. (2018). Simple and not‐so‐simple views of reading. Remedial and Special Education, 39(5), 313–316. 10.1177/0741932518770288 PMC6530938 31130774

[jcv212177-bib-0048] Snowling, M. J. (2008). Specific disorders and broader phenotypes: The case of dyslexia. Quarterly Journal of Experimental Psychology, 61(1), 142–156. 10.1080/17470210701508830 18038345

[jcv212177-bib-0049] Snowling, M. J. , & Hulme, C. (2021). Annual research review: Reading disorders revisited – The critical importance of oral language. Journal of Child Psychology and Psychiatry, 62(5), 635–653. 10.1111/jcpp.13324 32956509

[jcv212177-bib-0050] Tong, X. , Deacon, S. H. , Kirby, J. R. , Cain, K. , & Parrila, R. (2011). Morphological awareness: A key to understanding poor reading comprehension in English. Journal of Educational Psychology, 103(3), 523–534. 10.1037/a0023495

[jcv212177-bib-0051] Torppa, M. , Tolvanen, A. , Poikkeus, A.‐M. , Eklund, K. , Lerkkanen, M.‐K. , Leskinen, E. , & Lyytinen, H. (2007). Reading development subtypes and their early characteristics. Annals of Dyslexia, 57(1), 3–32. 10.1007/s11881-007-0003-0 17849214

[jcv212177-bib-0052] van den Akker, O. , Weston, S. J. , Campbell, L. , Chopik, W. J. , Damian, R. I. , Davis‐Kean, P. , Hall, A. , Kosie, J. , Kruse, E. , Olsen, J. , Ritchie, S. J. , Valentine, K. D. , Veer, A. van ’t , & Bakker, M. (2019). Preregistration of secondary data analysis: A template and tutorial. PsyArXiv. 10.31234/osf.io/hvfmr

[jcv212177-bib-0053] Vaughn, S. (2018). Introduction to the special issue on the simple view of reading from pre‐K to grade 12. Remedial and Special Education, 39(5), 259–259. 10.1177/0741932518772912

[jcv212177-bib-0054] Verhoeven, L. , Leeuwe, J. van , & Vermeer, A. (2011). Vocabulary growth and reading development across the elementary school years. Scientific Studies of Reading, 15(1), 8–25. 10.1080/10888438.2011.536125

[jcv212177-bib-0055] Wagner, R. K. , Beal, B. , Zirps, F. A. , & Spencer, M. (2021). A model‐based meta‐analytic examination of specific reading comprehension deficit: How prevalent is it and does the simple view of reading account for it? Annals of Dyslexia, 71(2), 260–281. 10.1007/s11881-021-00232-2 34080138 PMC8483584

[jcv212177-bib-0056] Wechsler, D. , Golombok, S. , & Rust, J. (1992). The wechsler intelligence scale for children—third edition UK manual. The Psychological Corporation.

[jcv212177-bib-0057] Weller, B. E. , Bowen, N. K. , & Faubert, S. J. (2020). Latent class analysis: A guide to best practice. Journal of Black Psychology, 46(4), 287–311. 10.1177/0095798420930932

