# Supplementary material for: Heterogeneity in children's reading comprehension difficulties: A latent class approach
Source: JCPP Adv. 2023 Jun 5;3(4):e12177. doi: 10.1002/jcv2.12177 (PMC10694533; doi:10.1002/jcv2.12177)
Supplement: Supplementary file 1 — Supplementary Material [file JCV2-3-e12177-s001.docx]

Supplemental Material for

*Heterogeneity in children’s reading comprehension difficulties: a latent class approach*

James, Thompson, Bowes, & Nation (2022)

**Index**

[Appendix S1 2](#_Toc135233289)

[Missing data 2](#_Toc135233290)

[Table S1. Missingness in variables planned for analysis 3](#_Toc135233291)

[Appendix S2 4](#_Toc135233292)

[Supplementary analysis details for RQ1 4](#_Toc135233293)

[Model fitting procedure 4](#_Toc135233294)

[Cross-validation 4](#_Toc135233295)

[Table S2. Fit indices for all pre-registered LPA models considered for Q1 5](#_Toc135233296)

[Figure S1. Elbow plots of fit indices for all pre-registered models (Q1) 7](#_Toc135233297)

[Appendix S3 1](#_Toc135233298)

[Supplementary analysis details for RQ2 1](#_Toc135233299)

[Factor structure 1](#_Toc135233300)

[Model simplification to ameliorate fit issues 1](#_Toc135233301)

[Figure S2. Weak comprehender performance on the decoding and comprehension measures 3](#_Toc135233302)

[Appendix S4 4](#_Toc135233303)

[Index for further model information available online 4](#_Toc135233304)

[References 5](#_Toc135233305)

# Appendix S1

## Missing data

We did not anticipate structural missingness in the data given that our subsample is defined by data at a later point in the study (age 9) and that the majority of the measures were collected relatively close in time (age 8-10). However, approximately 10-15% of the subsample were missing data from measures collected in different data collection waves. The vast majority of the missingness was at the level of the data collection wave—i.e., participants did not attend a particular clinic day, or return a particular questionnaire (Table S1). We thus tested whether missingness for each wave was predicted by each of the following: the selected measures described above, key demographic variables used to describe attrition in the ALSPAC sample (sex, ethnicity, maternal education, housing tenure, maternal age at birth, gestation, and birthweight), and preschool vocabulary ability (as an earlier measure of language ability, relevant to our key research questions). Using binomial regressions to predict missingness for each wave, we found that significant continuous predictors included age at other clinic visits and NARA comprehension score (both already incorporated in the planned analyses), and maternal age at birth. Associations with categorical variables were tested separately using chi-squared tests, finding that ethnicity, maternal education, and housing tenure were associated with clinic attendance and questionnaire completion—as has been documented by ALSPAC in the whole sample.

We pre-registered that missingness would be handled using full information maximum likelihood estimation, as is appropriate for data that are at least missing at random (i.e., missingness can be predicted by other observed variables). This method of estimation incorporates all of the available information for each part of the model, resulting in unbiased estimates providing that correlates of missingness are included in the model (Little et al., 2014). Given that there are not readily available options for incorporating auxiliary variables into mixture models, we tested whether the socio-demographic variables identified above provided any additional information about missingness over and above the age and comprehension variables already included in the analysis. The difference in model fit was not statistically significant (p = .65), supporting the assumption that the data are missing at random conditional on observed variables in our planned analyses. We thus proceeded to use full information maximum likelihood estimation as planned.

## Table S1. Missingness in variables planned for analysis

| Data collection wave  (% missing) | Measure | Data availability (n) | | Total missing  (%) | Measure-specific missing (%) |
| --- | --- | --- | --- | --- | --- |
| Focus @ 8  (13.75%) | TEACh Attentional control^1^ | 5758 | 15.89 | | 2.14 |
|  | TEACh Divided attention^1^ | 5652 | 17.44 | | 3.69 |
|  | TEACh Selective attention^1^ | 5742 | 16.13 | | 2.38 |
|  | WISC Block design^2^ | 5791 | 15.41 | | 1.66 |
|  | WISC Coding^2^ | 5856 | 14.46 | | 0.71 |
|  | WISC Object assembly^2^ | 5540 | 19.08 | | 5.33 |
|  | WISC Picture arrangement^2^ | 5797 | 15.32 | | 1.57 |
|  | WISC Picture completion^2^ | 5828 | 14.87 | | 1.12 |
|  | WISC Backward digit span | 5731 | 16.29 | | 2.54 |
|  | WISC Vocabulary | 5828 | 14.87 | | 1.12 |
|  | WOLD Listening comprehension | 5852 | 14.52 | | 0.77 |
|  | WOLD Vocabulary | 5828 | 14.87 | | 1.12 |
| Questionnaire KU (13.89%) | SDQ Hyperactivity subscale | 5812 | 15.10 | | 1.21 |
| Focus @ 9  (0.00%) | NARA Accuracy | 6846 | - | | - |
|  | NARA Comprehension | 6846 | - | | - |
|  | NARA Rate | 6828 | 0.26 | | 0.26 |
|  | Nonword reading^3^ | 6807 | 0.57 | | 0.57 |
|  | Word reading^3^ | 6816 | 0.44 | | 0.44 |
| Focus @ 10 (9.76%) | Counting span^1^ | 5855 | 14.48 | | 4.72 |

*Note.* 1. Measures excluded from final analysis due to model fit issues; 2. Measures used to form composite Performance IQ score; 3. Measures summed to form single item accuracy score.

# Appendix S2

## Supplementary analysis details for RQ1

### Model fitting procedure

We started by fitting 1-6 *k*-class models for each specification, and added models up to 10-classes given preliminary evidence that additional classes could yet improve model fit. We fitted each model using 500 random sets of starting values and 50 final stage optimizations in the first instance, and increased these values if the best loglikelihood value was not replicated (Lubke & Luningham, 2017). Similarly, if the solution reached by the adjusted LRT tests for the *k*+1 model differed from that reached by the initial model fit, then the model was re-fitted using increased starts and/or a more gentle perturbation of start values to ensure the best loglikelihood value was reached (Asparouhov & Muthen, 2019). Thus, we can be reasonably confident that the presented models do not reflect local maxima.

### Cross-validation

Given the exploratory nature of class enumeration (and the addition of non-preregistered models into the analysis), we sought to cross-validate the final model using the second half of the dataset. We followed the procedure set out by Masyn (2013). In the first step, we extracted the model parameters from our final model (the modified LPA with covariate), and fixed them in fitting a model to the second half of the dataset. A second model was then fitted of the same specification, allowing the parameters to be freely estimated. Ideally, these two models would show equivalently good fit to the data to indicate stability of profiles that are not over-fitted to nuances in the data. A nested LRT indicated a significant difference in fit between the two models (p < .001). However, an inspection of the classes in the freely estimated model suggested that this difference was largely driven by slight differences in the intercepts of each class, whereas the overall shape of the profiles remained similar to those identified in the initial model.

To be sure that we had arrived at the best model, we proceeded to carry out double cross-half validation: we re-ran the class enumeration process for the shape models using the second half of the sample. As above, the modified LPA analyses were clearly a better fit to the data than the factor mixture alternative. Although the LMR tests favoured the 5-class model, the 6-class model showed much better profile stability across the two halves of the sample, and thus was decided as the best model overall. The remaining results are based on the 6-class latent profile model that includes general ability as a covariate, re-fitted to the full dataset.

## Table S2. Fit indices for all pre-registered LPA models considered for Q1

| Specification | k | *n*  params | LL | BIC | CAIC | AWE | VLMR_p | LMR_p | BF | cmP_k | cmP_  best |  |
| --- | --- | --- | --- | --- | --- | --- | --- | --- | --- | --- | --- | --- |
| Model A | 1 | 17 | -60382.9 | 120904.1 | 120921.1 | 120929.6 | - | - | - | <0.01 | - |  |
|  | 2 | 24 | -57767.6 | 115730.4 | 115754.4 | 115766.4 | <0.01 | <0.01 | >100 | <0.01 | - |  |
|  | 3 | 31 | -56707.6 | 113667.4 | 113698.4 | 113713.9 | <0.01 | <0.01 | >100 | <0.01 | - |  |
|  | 4 | 38 | -56171.2 | 112651.7 | 112689.7 | 112708.7 | <0.01 | <0.01 | >100 | <0.01 | - |  |
|  | 5 | 45 | -55766.1 | 111898.4 | 111943.4 | 111965.9 | <0.01 | <0.01 | >100 | <0.01 | 0 |  |
|  | 6 | 52 | -55493.3 | 111409.7 | 111461.7 | 111487.7 | 0.19 | 0.19 | >100 | <0.01 | - |  |
|  | 7 | 59 | -55281.6 | 111043.3 | 111102.3 | 111131.8 | 0.41 | 0.42 | >100 | <0.01 | - |  |
|  | 8 | 66 | -55099.1 | 110735.3 | 110801.3 | 110834.3 | 0.19 | 0.2 | >100 | <0.01 | - |  |
| Model B | 1 | 23 | -56151.3 | 112489.7 | 112512.7 | 112524.2 | - | - | - | <0.01 | - |  |
|  | 2 | 30 | -55222.5 | 110689.1 | 110719.1 | 110734.1 | <0.01 | <0.01 | >100 | <0.01 | - |  |
|  | 3 | 37 | -54995.7 | 110292.5 | 110329.5 | 110348 | <0.01 | <0.01 | >100 | <0.01 | - |  |
|  | 4 | 44 | -54802.7 | 109963.4 | 110007.4 | 110029.4 | <0.01 | <0.01 | >100 | <0.01 | - |  |
|  | 5 | 51 | -54670.9 | 109756.9 | 109807.9 | 109833.4 | - | - | >100 | <0.01 | 0 |  |
|  | 6 | 58 | -54588.4 | 109648.9 | 109706.9 | 109735.9 | 0.44 | 0.44 | >100 | <0.01 | - |  |
|  | 7 | 65 | -54521 | 109571.1 | 109636.1 | 109668.6 | 0.24 | 0.24 | >100 | <0.01 | - |  |
|  | 8 | 72 | -54457.3 | 109500.5 | 109572.5 | 109608.5 | 0.1 | 0.1 | >100 | <0.01 | - |  |
| Model C | 1 | 17 | -60382.9 | 120904.1 | 120921.1 | 120929.6 | - | - | - | <0.01 | - |  |
|  | 2 | 28 | -57593.3 | 115414.4 | 115442.4 | 115456.4 | <0.01 | <0.01 | >100 | <0.01 | - |  |
|  | 3 | 39 | -56631.4 | 113580.2 | 113619.2 | 113638.7 | 0.01 | 0.01 | >100 | <0.01 | - |  |
|  | 4 | 50 | -55930 | 112266.9 | 112316.9 | 112341.9 | <0.01 | <0.01 | >100 | <0.01 | - |  |
|  | 5 | 61 | -55405.6 | 111307.6 | 111368.6 | 111399.1 | <0.01 | <0.01 | >100 | <0.01 | 0 |  |
|  | 6 | 72 | -55114.3 | 110814.6 | 110886.6 | 110922.6 | <0.01 | <0.01 | >100 | <0.01 | - |  |
|  | 7 | 83 | -54965.4 | 110606.3 | 110689.3 | 110730.8 | 0.08 | 0.08 | >100 | <0.01 | - |  |
|  | 8 | 94 | -54842 | 110449 | 110543 | 110590 | 0.05 | 0.05 | >100 | <0.01 | - |  |
| Model D | 1 | 23 | -56151.3 | 112489.7 | 112512.7 | 112524.2 | - | - | - | <0.01 | - |  |
|  | 2 | 34 | -55222.1 | 110720.9 | 110754.9 | 110771.9 | <0.01 | <0.01 | >100 | <0.01 | - |  |
|  | 3 | 45 | -54922.9 | 110212 | 110257 | 110279.5 | <0.01 | <0.01 | >100 | <0.01 | 0 |  |
|  | 4 | 56 | -54722.6 | 109901 | 109957 | 109985 | <0.01 | <0.01 | >100 | <0.01 | - |  |
|  | 5 | 67 | -54558.1 | 109661.4 | 109728.4 | 109761.9 | <0.01 | <0.01 | >100 | <0.01 | - |  |
|  | 6 | 78 | -54469.9 | 109574.6 | 109652.6 | 109691.6 | 0.25 | 0.25 | >100 | <0.01 | - |  |
|  | 7 | 89 | -54373 | 109470.3 | 109559.3 | 109603.8 | 0.26 | 0.26 | >100 | <0.01 | - |  |
|  | 8 | 100 | -54288.9 | 109391.6 | 109491.6 | 109541.6 | 0.07 | 0.07 | >100 | <0.01 | - |  |
| Model E | 1 | 23 | -56151.3 | 112489.7 | 112512.7 | 112524.2 | - | - | - | <0.01 | - |  |
|  | 2 | 40 | -55212.9 | 110751.3 | 110791.3 | 110811.3 | <0.01 | <0.01 | >100 | <0.01 | - |  |
|  | 3 | 57 | -54753.1 | 109970 | 110027 | 110055.5 | <0.01 | <0.01 | >100 | <0.01 | - |  |
|  | 4 | 74 | -54528.5 | 109659.3 | 109733.3 | 109770.3 | <0.01 | <0.01 | >100 | <0.01 | 1 |  |
|  | 5 | 91 | -54325.8 | 109392.2 | 109483.2 | 109528.7 | 0.01 | 0.01 | >100 | <0.01 | - |  |
|  | 6 | 108 | -54154 | 109186.9 | 109294.9 | 109348.9 | <0.01 | <0.01 | >100 | <0.01 | - |  |
|  | 7 | 125 | -54075.4 | 109168.1 | 109293.1 | 109355.6 | 0.48 | 0.48 | >100 | >0.99 | - |  |
|  | 8 | 142 | -54036.3 | 109228.3 | 109370.3 | 109441.3 | - | - | <0.01 | <0.01 | - |  |
| *Note.* Model A = Class invariant, diagonal; Model B = Class invariant, unrestricted; Model C = Class-varying, diagonal; Model D = Class-varying (variances only), unrestricted; Model E = Class-varying (full), unrestricted. | | | | | | | | | | | | |

## Figure S1. Elbow plots of fit indices for all pre-registered models (Q1)


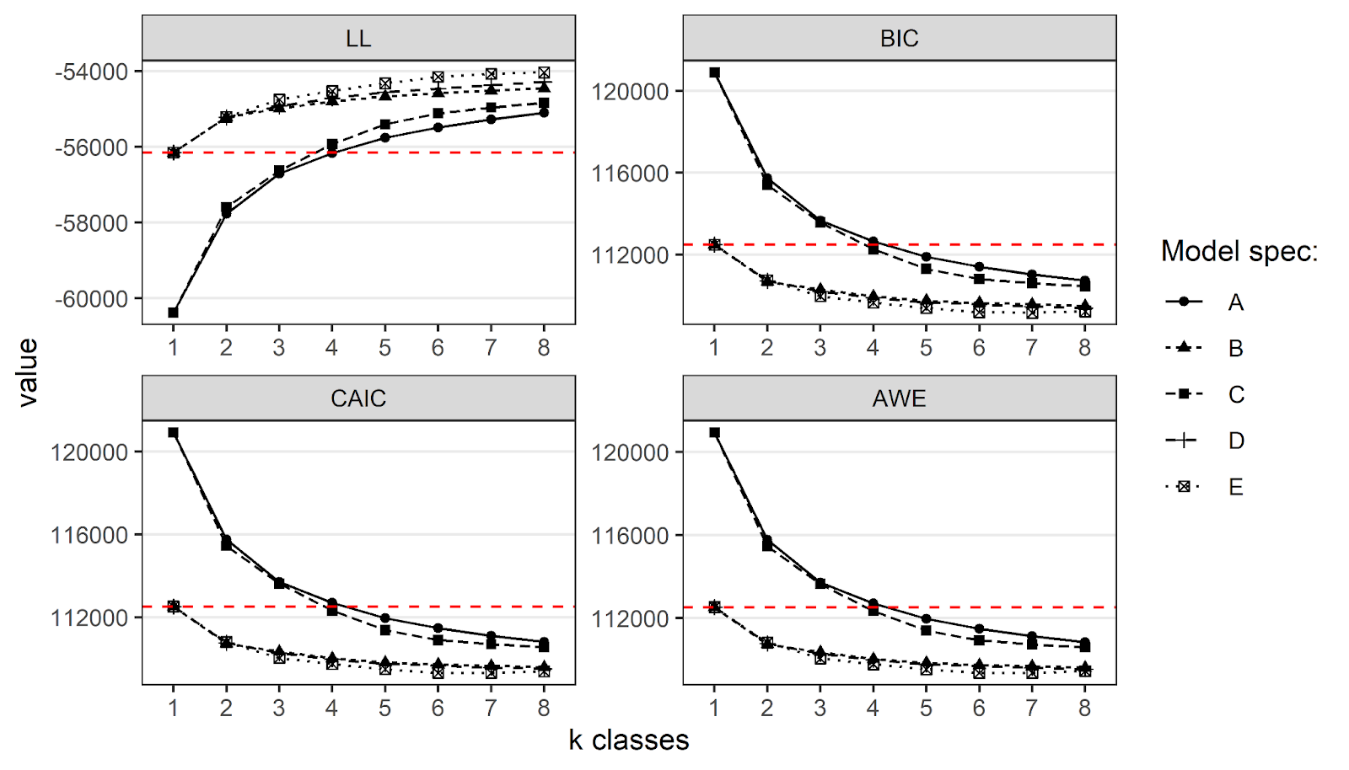


*Note.* Model A = Class invariant, diagonal; Model B = Class invariant, unrestricted; Model C = Class-varying, diagonal; Model D = Class-varying (variances only), unrestricted; Model E = Class-varying (full), unrestricted.

# Appendix S3

## Supplementary analysis details for RQ2

### Factor structure

As noted in the main manuscript, we originally intended to include additional measures of executive function: a counting span task (Case et al., 1982) and three attention measures from the Test of Everyday Attention for Children (TEACh; Manly et al., 2001). We ran an initial CFA to assess our proposed factor structure: decoding (item accuracy, passage accuracy), comprehension (reading comprehension, listening comprehension), vocabulary (picture naming, definitions), performance IQ (all five subtests), executive function (two working memory measures, three attention measures, behavioural inattention), and reading fluency (single observed variable). Model fit was poor, and modification indices indicated issues relating to the high correlation between passage accuracy and passage comprehension. We addressed this issue by incorporating a cross-loading between reading comprehension and the decoding latent variable, and further allowing for correlated errors between the two original decoding measures. The correlation between the comprehension and vocabulary latent constructs was also very high, consistent with the Simple View perspective that oral language skills are key for this component of reading. Thus, we combined these measures in  a single language construct (see Language and Reading Research Consortium, 2017, for similar findings). We were also guided by the modification indices to include a cross-loading between the WISC code subtest of the Performance IQ measures to executive function

### Model simplification to ameliorate fit issues

Although the resulting factor structure showed good overall model fit (CFI = 0.97; TLI = 0.96; RMSEA = 0.03; SRMR = 0.04) the model encountered convergence issues relating to model fit and complexity upon introduction of the latent class structure. This was particularly the case for constructs where item loadings had been weak: for the attention variables, and performance IQ. The model was respecified following indicators of the source of the fit issues within the Mplus output. Specifically, for the attention measures, the following respecifications were applied: incorporating measures separately; removing one measure; removing outliers; and transforming certain measures to address distributional issues, but no solution provided improved fit to the data. Given the weak correlations between each measure, a composite score was deemed inappropriate and these measures excluded from further analysis.

Once attention variables were removed, the initial executive function construct consisted of three categorical measures: two working memory measures, and behavioural inattention. The inclusion of all three assessments also led to model errors due to the number of empty cells in each class, and these could not be modelled as a latent construct. One working memory measure was selected and entered alongside behavioural inattention as observed variables (directly predicted by the latent class and no other latent construct).

For performance IQ, the subtests have well-established use as a combined assessment, and we did not have prior hypotheses regarding poor comprehenders’ performance on any particular subtest. Thus, the subtest scores were averaged into a single z-score composite to simplify the model and ameliorate errors. Following manual guidelines, a child had a composite performance IQ score if they completed a minimum of four of the five subtests.

In sum, the most complete model that we were able to specify for the final factor mixture analysis included latent variables for decoding (item accuracy, passage accuracy, passage comprehension) and language (listening comprehension, passage comprehension, picture naming, definitions), alongside four directly observed variables (reading fluency, working memory, behavioural inattention, performance IQ composite). This model still allowed us to consider all key constructs of interest, albeit in a reduced form.

## Figure S2. Weak comprehender performance on the decoding and comprehension measures


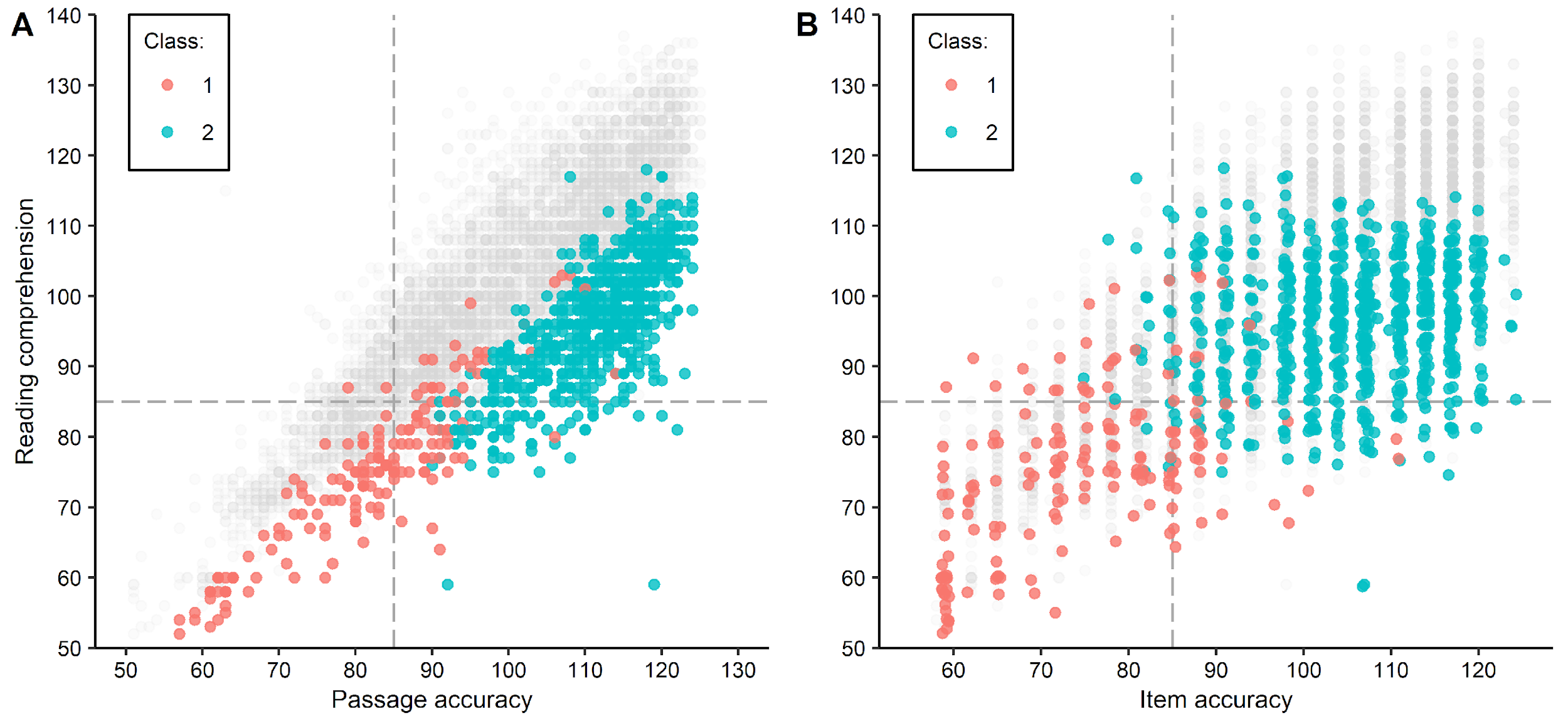


*Note.* A) Performance on the passage accuracy and comprehension measures of the NARA-II; B) Performance on the item accuracy and reading comprehension measures. The remainder of the sample (not included in the analyses for Q2) are plotted in grey for reference. The dashed lines mark a threshold often used to identify children as impaired on either task (standardised score < 85). Thus, traditional methods of identifying poor comprehenders would select children in the bottom right quadrant of either figure, with those further to the right most likely meeting additional criteria of a discrepancy between the two scores.

# Appendix S4

## Index for further model information available online

Further details and annotated analysis scripts are available online and can be accessed through the project page indexed first. Key links to final model information are provided here for ease of access.

| **Description** | **Direct link** |
| --- | --- |
| Project page | <https://doi.org/10.17605/OSF.IO/ZVJW4> |
| Preregistration | <https://osf.io/awqs2> |
| Analysis 1A: Final model (html) | <https://osf.io/2mjqy> |
| Analysis 1A: Final model (Mplus) | <https://osf.io/234ke> |
| Analysis 1B: Final model (html) | <https://osf.io/dy4j2> |
| Analysis 1B: Final model (Mplus output) | <https://osf.io/ybzg3> |
| Analysis 2: Final model (html) | <https://osf.io/fcbv5> |
| Analysis 2: Final model (Mplus output) | <https://osf.io/39dac> |

# References

Asparouhov, T., & Muthen, B. (2019). *Random Starting Values and Multistage Optimization*. 8.

Case, R., Kurland, D. M., & Goldberg, J. (1982). Operational efficiency and the growth of short-term memory span. *Journal of Experimental Child Psychology*, *33*(3), 386–404.

Language and Reading Research Consortium. (2017). Oral Language and Listening Comprehension: Same or Different Constructs? *Journal of Speech, Language, and Hearing Research*, *60*(5), 1273–1284. https://doi.org/10.1044/2017_JSLHR-L-16-0039

Little, T. D., Jorgensen, T. D., Lang, K. M., & Moore, E. W. G. (2014). On the Joys of Missing Data. *Journal of Pediatric Psychology*, *39*(2), 151–162. https://doi.org/10.1093/jpepsy/jst048

Lubke, G. H., & Luningham, J. (2017). Fitting latent variable mixture models. *Behaviour Research and Therapy*, *98*, 91–102. https://doi.org/10.1016/j.brat.2017.04.003

Manly, T., Anderson, V., Nimmo‐Smith, I., Turner, A., Watson, P., & Robertson, I. H. (2001). The Differential Assessment of Children’s Attention: The Test of Everyday Attention for Children (TEA-Ch), Normative Sample and ADHD Performance. *Journal of Child Psychology and Psychiatry*, *42*(8), 1065–1081. https://doi.org/10.1111/1469-7610.00806

Masyn, K. E. (2013). 25 latent class analysis and finite mixture modeling. *The Oxford Handbook of Quantitative Methods*, 551.
